# The role of neutrophil-to-lymphocyte ratio in the prognosis of chronic kidney disease: insights from the NHANES cohort study

**DOI:** 10.3389/fsysb.2025.1656683

**Published:** 2025-10-27

**Authors:** Ying Liu, Ru Wang, Jinguo Yuan, Jin Zhao

**Affiliations:** ^1^ Military Personnel Medical Center, 986th Hospital of PLAAF, Fourth Military Medical University, Xi’an, Shaanxi, China; ^2^ Department of Clinical Skills Training Center, Xijing Hospital, Fourth Military Medical University, Xi’an, China; ^3^ Department of Nephrology, Xijing Hospital, Fourth Military Medical University, Xi’an, Shaanxi, China

**Keywords:** neutrophil-to-lymphocyte ratio (NLR), chronic kidney disease (CKD), all-cause mortality, cardiovascular disease mortality, inflammation

## Abstract

**Objective:**

To investigate the association of neutrophil-to-lymphocyte ratio (NLR) with the cardiovascular disease (CVD) and all-cause mortality in patients with chronic kidney disease (CKD).

**Methods:**

Using date from NHANES survey 2009–2018, 2,635 patients with CKD were eventually included in this study. The population was stratified into two groups based on the median NLR. Kaplan-Meier method with log-rank tests for significance was used for survival analysis. Weighted Cox proportional hazards regression models were employed to estimate the hazard ratio (HR) and corresponding 95% confidence interval (CI) for all-cause and CVD mortality. The potential nonlinear relationship between NLR and CVD and all-cause mortality was assessed using restricted cubic spline (RCS) models. The time-dependent receiver operating characteristic (ROC) curve was utilized to assess the precision of NLR in predicting survival outcomes.

**Results:**

The Kaplan-Meier curve indicated a significant difference in overall survival between the two groups (log-rank test, p < 0.0001). Compared to lower NLR group, participants in the higher NLR group had HR of 1.56 (1.30, 1.87) for all-cause mortality and 2.07 (1.51, 2.84) for CVD mortality, respectively. We observed a significant nonlinear relationship between NLR and CVD and all-cause mortality (p < 0.0001). The time-dependent ROC curve demonstrated that the areas under the curve for 1-, 3-, 5-, and 10-year survival rates were 0.69, 0.65, 0.63, and 0.62 for all-cause mortality, and 0.71, 0.67, 0.66, and 0.64 for CVD mortality, respectively.

**Conclusion:**

A higher NLR is linked to an elevated risk of CVD and all-cause mortality in patients with CKD. Additionally, NLR can serve as a potential prognostic indicator for CKD patients.

## Introduction

Chronic kidney disease (CKD) is characterized by the progressive and irreversible loss of renal function, which can ultimately lead to end-stage renal disease (ESRD) and premature death (Mihai et al., 2019). It has emerged as a significant public health concern, affecting over 9.1% of the global population and resulting in approximately 1.2 million deaths annually (Global, 2020). Particularly, patients with CKD face an elevated risk of cardiovascular disease (CVD) (Koga et al., 2022), a common comorbidity that is responsible for nearly half of all deaths among individuals with CKD (Ferrè et al., 2023). Therefore, the identification of reliable biomarkers is crucial in predicting cardiovascular events and mortality risk in CKD patients.

The understanding of the pathological mechanisms underlying CKD has expanded over the past decade, revealing a crucial role of immune cells in disease initiation and progression (Yan et al., 2023). Among the various immune cells involved in CKD, neutrophils and lymphocytes have gained particular attention due to their significant correlation with inflammatory processes that occur in CKD. Neutrophils are key innate immune cells that protect the host against invading pathogens and sterile tissue damage (Lucas et al., 2013). However, their excessive activation or prolonged retention in the circulation can lead to tissue damage and the progression of various diseases, including CKD (Gao et al., 2016). Lymphocytes, on the other hand, are crucial for immune regulation and tissue repair (Gasteiger et al., 2013). Dysfunctional lymphocytes have been reported in CKD patients, contributing to the development of tubulointerstitial inflammation and fibrosis (Cao et al., 2021).

The neutrophil-to-lymphocyte ratio (NLR) has been proposed as a simple and cost-effective biomarker, offering valuable insights into the intricate balance between inflammation and immune response. NLR has emerged as a novel survival indicator in various diseases, including CVD (Dentali et al., 2018), cancer (Byrne et al., 2022), and infectious diseases (Zhang et al., 2023). Recently, elevated NLR has also been shown to be associated with increased risk of CVD events and mortality in patients with end stage-CKD and dialysis patients (Lano et al., 2022; Yuan et al., 2019). However, it remains unclear whether NLR can serve as a prognostic indicator for general CKD patients.

Our research endeavors to offer fresh perspectives on the intricate link between NLR and both all-cause and CVD mortality among CKD patients in the United States. To achieve this, we conducted a retrospective cohort study, leveraging the comprehensive data available in the National Health and Nutrition Examination Surveys (NHANES) database.

## Methods

### Data sources and study population

The NHANES, a continuous and comprehensive cross-sectional study conducted in the United States, employs an intricate and meticulous multistage sampling methodology to gain insights into the health status and nutritional profile of the American population (Inoue et al., 2020). This survey, which collects data from interviews, physical examinations, and laboratory tests, releases its findings every 2 years, providing a valuable snapshot of the nation’s health trends over time (Sellmayr et al., 2020). The rigorous study protocols of NHANES have been approved by the Ethics Review Board of the National Center for Health Statistics, and all participants have provided their written informed consent, ensuring the ethical integrity of the research.

For this study, we amalgamated data from five consecutive NHANES cycles (spanning from 2009 to 2018) with mortality data from the National Death Index. Utilizing the Chronic Kidney Disease Epidemiology Collaboration (CKD-EPI) equation, we calculated the estimated glomerular filtration rate (eGFR) for each participant based on their baseline creatinine levels (Levey and Stevens, 2010), and defined CKD as an eGFR of less than 60 mL/min/1.73 m^2^. To ensure the accuracy and reliability of our analysis, we excluded individuals under the age of 18 or those without information on neutrophil count, lymphocyte count, creatinine levels, or death status. The included individuals were stratified into two groups based on the median NLR: group 1 comprising patients below the median NLR value and group 2 comprising those above it.

### Exposure measurement

The NHANES database served as a valuable resource for obtaining the complete blood count (CBC) profile, including neutrophil and lymphocyte counts. A single blood sample was collected from each participant at the time of enrollment, ensuring consistency and reliability in the data. The NHANES website offers extensive information on the laboratory methodologies employed, rigorous quality assurance measures undertaken, and sophisticated data processing techniques implemented, thereby enhancing the credibility and accuracy of the CBC data. NLR was calculated for each participant by dividing the total absolute neutrophil counts by the total absolute lymphocyte counts.

### Outcome ascertainment

The primary objectives of this study were to investigate two key outcomes: all-cause mortality and cardiovascular disease (CVD) mortality. To gather mortality data up until 31 December 2019, we utilized the NHANES Public-Use Linked Mortality File, which is seamlessly integrated with the National Death Index. We relied on the International Statistical Classification of Diseases, 10th Revision (ICD-10), to accurately determine the underlying cause of death for each participant. Specifically, CVD mortality encompassed deaths attributed to heart diseases (ICD-10 codes I00-I09, I11, I13, I20-I51) and cerebrovascular disease (ICD-10 codes I60-I69). For each participant, we calculated the duration of the event, which was defined as the time elapsed from their visit to the NHANES Mobile Examination Center (MEC) until their last known alive date or the date of censoring from the mortality file.

### Covariates

This study examined various factors, including demographics, physical exam results, and health conditions. Demographics like gender, race, BMI, income, and education were categorized. Alcohol consumption was split into abstainers, moderate, and heavy drinkers. Smoking status was classified as nonsmokers, former, and current smokers. Diabetes was identified by diagnosis, HbA1c levels, or fasting plasma glucose concentrations. Hypertension was diagnosed based on medication use or blood pressure measurements. Dyslipidemia was defined by diagnosis, medication use, triglyceride levels, or HDL cholesterol levels.

### Statistical analysis

To ensure the representativeness of our analysis for the United States population, we applied the complex sampling design of NHANES by incorporating appropriate sample weights. We categorized continuous variables, excluding age, neutrophil count, lymphocyte count, and eGFR, and handled missing values as a distinct category. Descriptive statistics were calculated using mean and standard error for continuous variables and weighted proportions for categorical variables, within groups. Baseline characteristic differences were assessed using Mann-Whitney U-test and weighted chi-square tests for continuous and categorical variables, respectively. The Kaplan-Meier survival curves with log-rank tests were employed for survival analysis. To estimate hazard ratios for all-cause and CVD mortality, we utilized weighted multivariable Cox proportional hazards regression models, adjusting for potential confounders across four models. Restricted cubic spline models were used to identify potential non-linear associations between NLR and mortality outcomes, with gender- and age-specific analyses conducted. Stratified analyses and interaction analyses were performed to investigate potential modifiers and the interplay between NLR and stratified variables. Sensitivity analyses were conducted by excluding specific patient subsets, and time-dependent ROC curves were evaluated to assess the predictive accuracy of NLR. All statistical analyses were conducted using R software, with statistical significance at P < 0.05.

## Results

### Patient baseline characteristics

**FIGURE 1:**
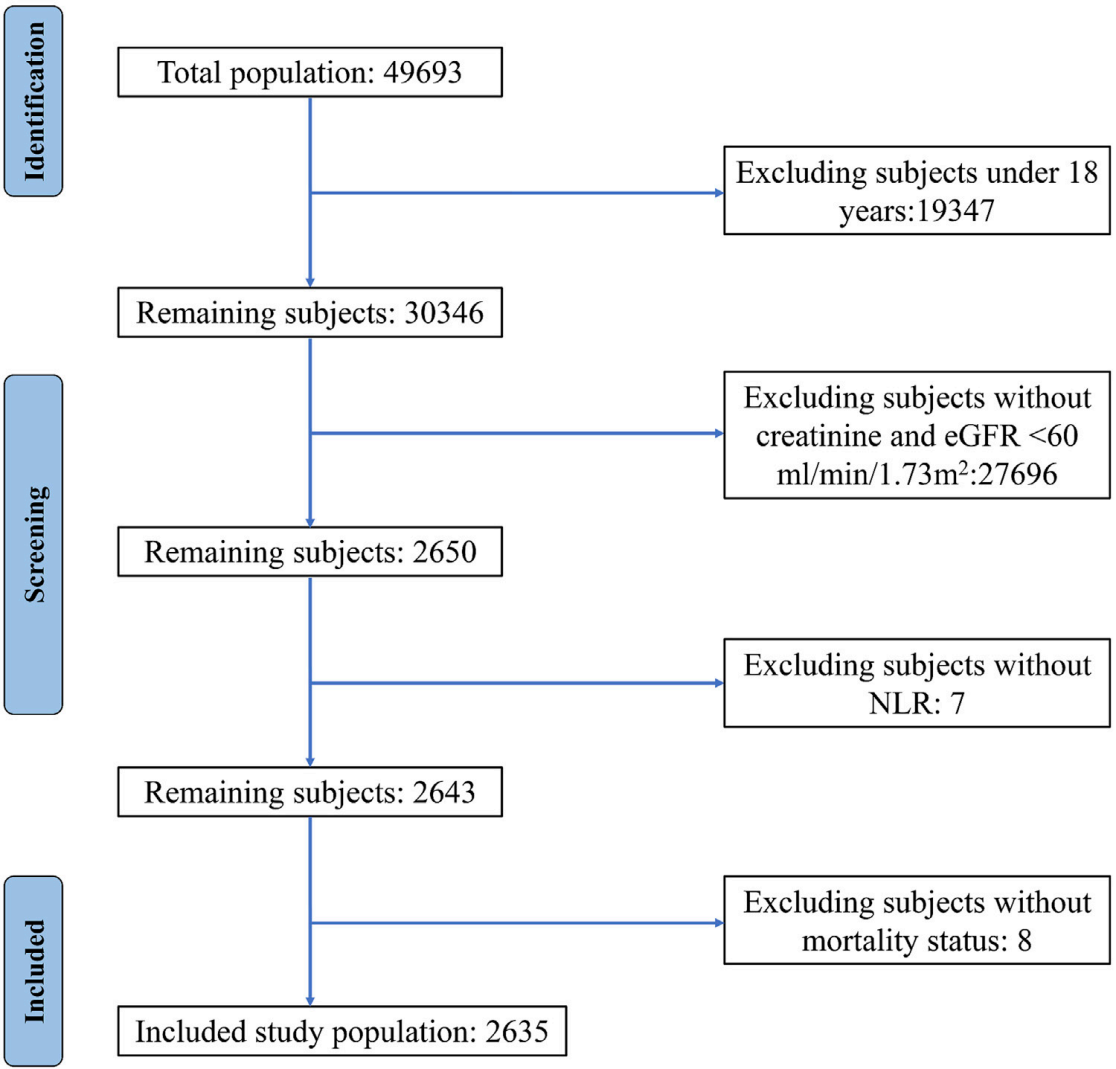
Flow chart of the study population selection.

A total of 2,635 patients, consisting of 1,305 males and 1,330 females, were included in this study (Figure 1). The mean age of the participants was 69.8 years, and the median NLR value was 2.23, with the cohort divided into two groups of 1,319 and 1,316 individuals, respectively. During a mean follow-up period of 60 months, 758 deaths were recorded, including 277 deaths attributed to CVD. Notably, the two groups experienced 280 and 478 deaths, respectively. The baseline demographic and clinical characteristics of the patients are summarized in Table 1.

**TABLE 1:** Baseline characteristics of participants with CKD in the NHANES 2009–2018 cohort.

		NLR		
Characteristic	Overall	≤2.23	>2.23	*P value*
Participants, n	2635	1319	1316	
Gender, %				<0.0001
Male	1305 (42.6%)	597 (38.1%)	708 (47.0%)	
Female	1330 (57.31%)	722 (61.9%)	608 (53.0%)	
Age, years	69.8 (0.28)	68.4 (0.42)	71.2 (0.38)	<0.0001
Race or ethnicity, %				
Non-Hispanic White	1208 (67.3%)	510 (61.6%)	698 (72.7%)	<0.0001
Non-Hispanic Black	790 (16.7%)	506 (22.3%)	284 (11.5%)	
Mexican American or Hispanic	447 (10.7%)	204 (10.0%)	243 (11.3%)	
Other	190 (5.3%)	99 (6.1%)	91 (4.5%)	
BMI, kg/m2, %				0.0003
<25.0	549 (21.1%)	255 (20.4%)	294 (22.31%)	
25.0–29.9	861 (32.2%)	446 (32.8%)	415 (35.74%)	
≥30.0	1149 (44.0%)	597 (45.5%)	552 (41.96%)	
Missing data	76 (2.7%)	21 (1.3%)	55 (3.9%)	
Education level, %				0.4960
Less than high school	755 (20.4%)	371 (20.2%)	384 (20.6%)	
High school	645 (25.6%)	327 (24.5%)	318 (26.7%)	
College or higher	1227 (53.8%)	617 (55.1%)	610 (52.6%)	
Missing data	8 (0.2%)	4 (0.2%)	4 (0.1%)	
Ratio of family income to poverty, %				
≤1.30	752 (21.0%)	401 (22.1%)	351 (19.9%)	0.0024
1.31–3.50	1028 (39.0%)	474 (35.9%)	554 (41.9%)	
>3.50	607 (32.2%)	327 (34.8%)	280 (29.8%)	
Missing data	248 (7.8%)	117 (7.1%)	131 (8.4%)	
Alcohol, %				<0.0001
None	780 (27.9%)	401 (28.7%)	379 (27.0%)	
Moderate	727 (32.4%)	343 (31.0%)	384 (33.7%)	
Heavy	319 (13.1%)	185 (15.9%)	134 (10.6%)	
Missing data	809 (26.6%)	390 (24.4%)	419 (28.7%)	
Smoke, %				0.0010
Nonsmoker	1260 (50.2%)	668 (53.5%)	592 (47.1%)	
Former smoker	1038 (39.2%)	467 (35.3%)	571 (42.9%)	
Current smoker	335 (10.5%)	183 (11.1%)	152 (9.9%)	
Missing data	2 (0.1%)	1 (0.1)	1 (0.1%)	
Diabetes, %	979 (32.9%)	428 (26.3%)	551 (39.2%)	<0.0001
Hypertension, %	2269 (83.9%)	1,130 (65.3%)	1,139 (68.4%)	<0.0001
Dyslipidemia, %	1750 (67.0%)	862(65.3%)	888 (68.4%)	0.0952
Neutrophils number (1000 cell/uL)	4.47 (0.04)	3.66 (1.30)	5.24 (2.10)	<0.0001
Lymphocyte number (1000 cells/uL)	2.14 (0.10)	2.74 (0.20)	1.57 (0.02)	<0.0001
NLR	2.61 (0.04)	1.61 (0.01)	3.55 (0.05)	<0.0001
eGFR, mL/min/1.73 m^2^	47.3 (0.28)	49.23 (0.34)	45.57 (0.43)	<0.0001
45-49	1,723 (65.39%)	936 (70.96%)	787 (59.80%)	
30-44	638 (24.21%)	296 (22.44%)	342 (25.99%)	
15-29	175 (6.64%)	62 (4.70%)	113 (8.59%)	
<15	99 (3.76%)	25 (1.90%)	74 (5.62%)	
All-cause mortality, %	758 (25.5%)	280 (18.9%)	478 (31.8%)	<0.0001
CVD-cause mortality, %	277 (10.5%)	88 (5.6%)	189 (12.8%)	<0.0001
Follow time, months	60.0 (0.28)	63.2 (34.0)	56.9 (33.4)	<0.0001

Values are weighted mean (SE) for continuous variables or numbers (weighted %) for categorical variables. CKD, chronic kidney disease; BMI, body mass index; NLR, Neutrophil-to-lymphocyte ratio; eGFR, estimated glomerular filtration rate; CVD, cardiovascular disease; NHANES, National Health and Nutritional Examination Survey.

### All-cause and CVD mortality

Table 2 presents the unadjusted and multivariable-adjusted hazard ratio (HR) with 95% confidence intervals (CIs) for all-cause and CVD mortality, exploring the influence of baseline NLR as both a continuous and categorical variable. When NLR is analyzed as a continuous variable, each unit increase is associated with an elevated HR of 1.04 (1.02, 1.14) for all-cause mortality and 1.10 (1.04, 1.16) for CVD mortality in the fully adjusted model. When NLR is categorized, participants in group 2, compared to the reference group 1, exhibit an HR of 1.56 (1.30, 1.87) for all-cause mortality and 2.07 (1.51, 2.84) for CVD mortality. Notably, the association between baseline NLR levels and mortality outcomes is slightly attenuated after adjustment for potential confounders.

**TABLE 2:** Associations of NLR with all-cause and CVD mortality in patients with CKD from the NHANES 2009–2018 cohort.

		HR (95% CI)			
	No. of Events	Model 1	Model 2	Model 3	Model 4
All-cause mortality					
NLR (continuous)	758	1.11 (1.05, 1.16)	1.08 (1.02, 1.13)	1.09 (1.03, 1.14)	1.04 (1.02, 1.14)
NLR (categorical)					
NLR_1	280	1.0 (reference)	1.0 (reference)	1.0 (reference)	1.0 (reference)
NLR_2	478	1.90 (1.58, 2.29)	1.59 (1.33, 1.91)	1.56 (1.30,1.87)	1.56 (1.30,1.87)
CVD mortality					
NLR (continuous)	277	1.11 (1.06, 1.18)	1.09 (1.03, 1.15)	1.10 (1.04, 1.16)	1.10 (1.04, 1.16)
NLR (categorical)					
NLR_1	88	1.0 (reference)	1.0 (reference)	1.0 (reference)	1.0 (reference)
NLR_2	189	2.55 (1.88,3.46)	2.13 (1.56,2.91)	2.06 (1.50,2.83)	2.07 (1.51,2.84)

Values are n or weighted HR (95% CI). Model 1 is unadjusted; Model 2 is adjusted for: Age, Sex and Race; Model 3 is adjusted for: model 2 + Alcohol intake, Smoking status, BMI, Ratio of family income to poverty, and Education level; Model 4 is adjusted for: model 3 plus Diabetes, Hypertension, Dyslipidemia and eGFR; CVD, cardiovascular disease; CKD, chronic kidney disease, NHANES, National Health and Nutritional Examination Survey; NLR, Neutrophil-to-lymphocyte ratio.

### Kaplan-Meier survival curve analysis

**FIGURE 2:**
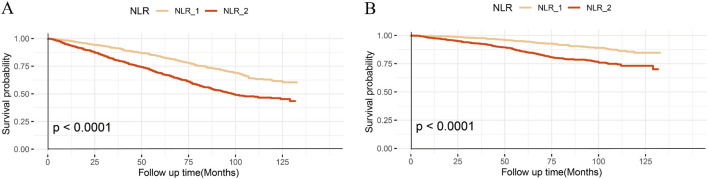
Kaplan–Meier curves of the survival rate with higher and lower NLR groups. **(A)**, All-cause mortality; **(B)**, CVD mortality.

The Kaplan-Meier curve illustrates a notable difference in the overall survival rates when considering all-cause (Figure 2A) and CVD mortality (Figure 2B) between the two groups (log-rank test, p < 0.0001).

### Smoothing curve fitting

**FIGURE 3:**
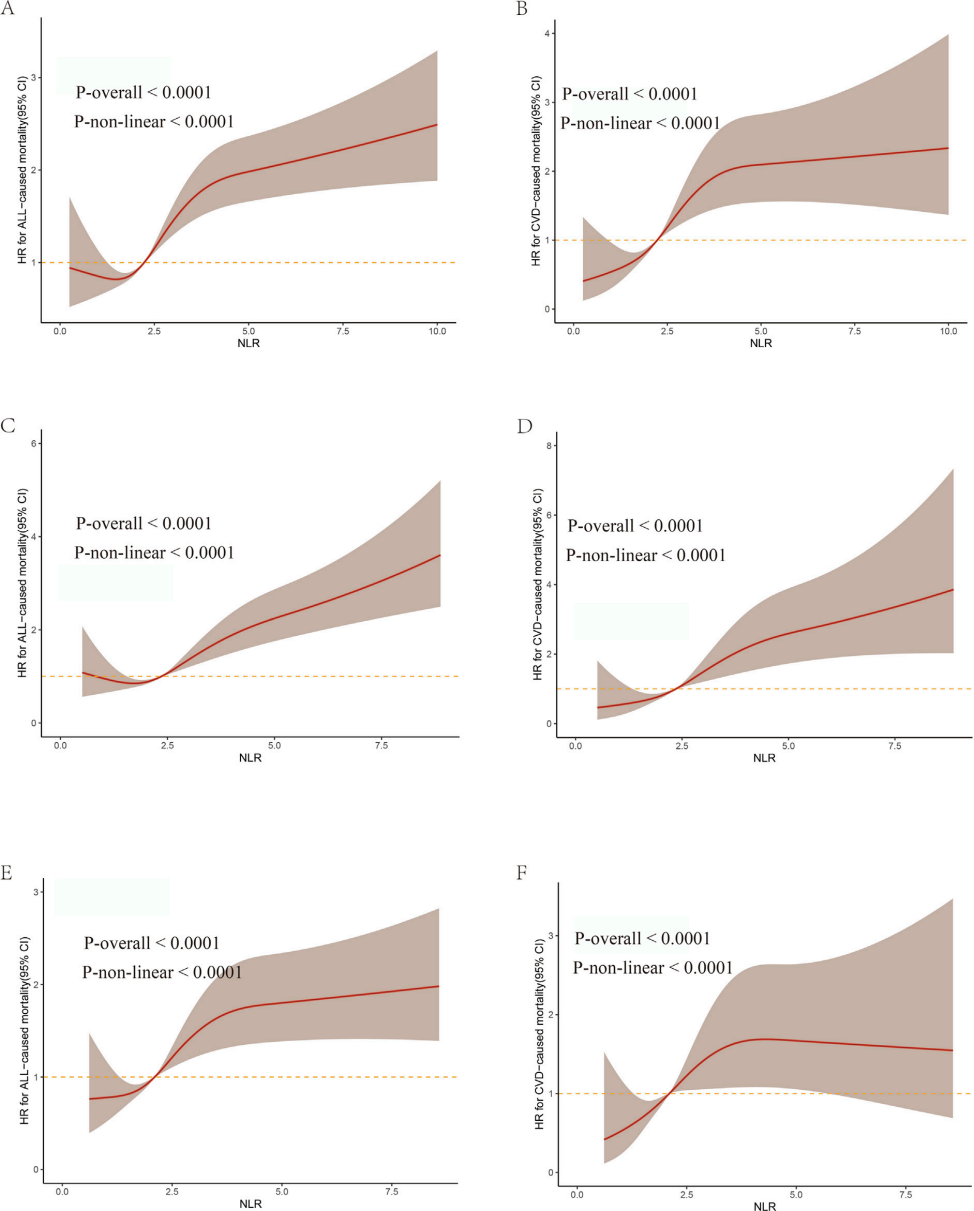
The weighted restricted cubic splines for associations of NLR levels with all-cause and CVD mortality from NHANES 2009–2018. **(A,C,E)** represent all-cause mortality in all participants, males, and females respectively. **(B,D,F)** represent CVD mortality in all participants, males, and females, respectively.

We identified a significant nonlinear relationship between the NLR and both all-cause CVD mortality, which was consistent across the entire population and gender, with all p values for non-linear <0.0001 (Figures 3A–F). As the NLR value increases, there is an elevated risk for both all-cause and CVD mortality in all participants, especially with a significantly steeper trend in males.

### Subgroup analysis

**FIGURE 4:**
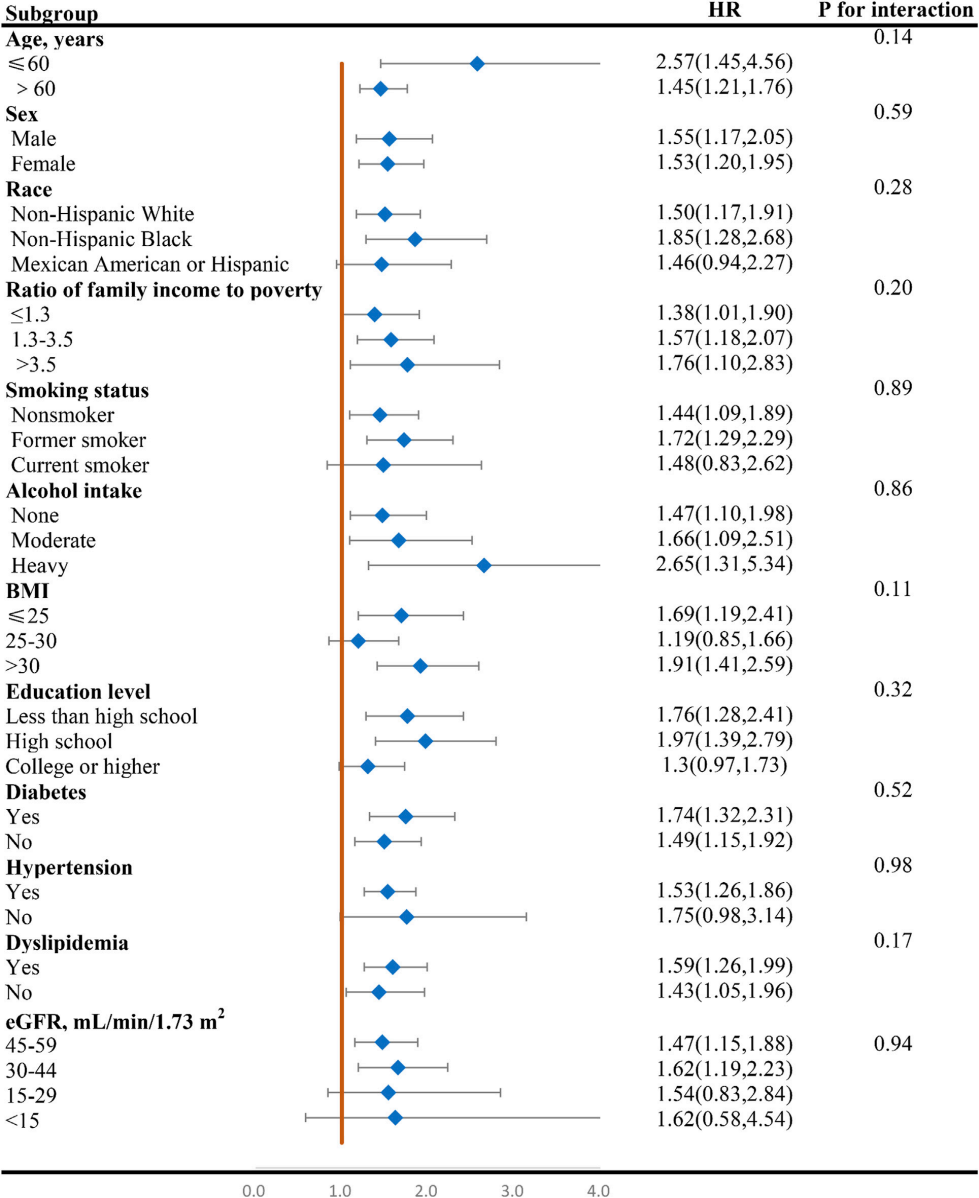
Forest plot of HR of higher NLR group compared with lower NLR group for all-cause mortality in patients with CKD. NLR, Neutrophil-to-lymphocyte ratio; HR, hazard ratio; CKD, chronic kidney disease; BMI, body mass index; eGFR, estimated glomerular filtration rate.

**FIGURE 5:**
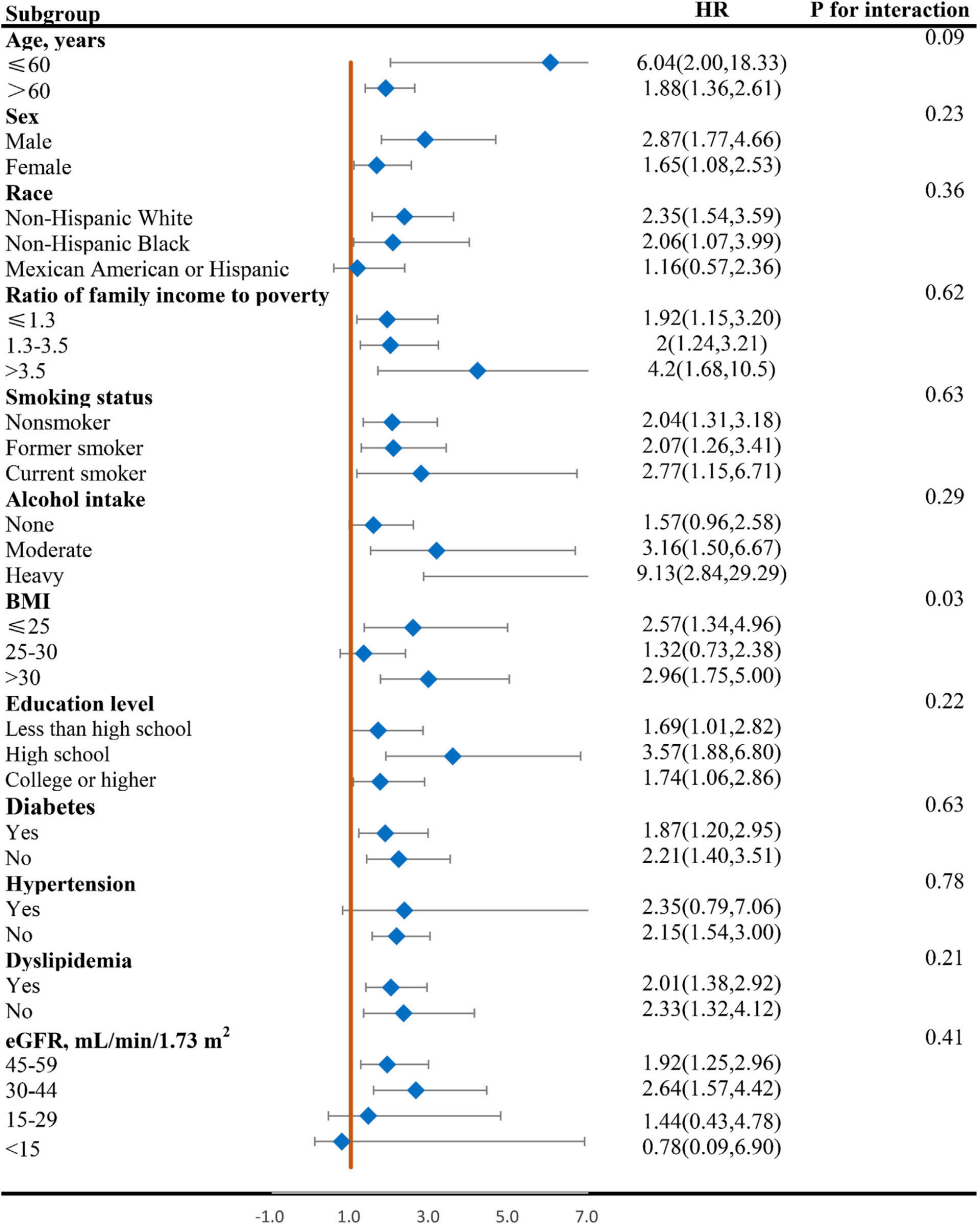
Forest plot of HR of higher NLR group compared with lower NLR group for CVD mortality in patients with CKD. NLR, Neutrophil-to-lymphocyte ratio; HR, hazard ratio; CVD, cardiovascular disease; CKD, chronic kidney disease; BMI, body mass index; eGFR, estimated glomerular filtration rate.

Figures 4, 5 display the correlation between NLR and the risk of long-term all-cause and CVD mortality in various subgroups. The findings largely align with preliminary results, except for a few subgroups where statistical significance was not achieved. The interaction analysis revealed a significant interaction between BMI and NLR during cardiovascular mortality (p = 0.03). Regarding different CKD stages, NLR significantly increased the all-cause and CVD death risk for G3a and G3b, while the result of G4 and G5 phases were not statistically significant due to the small sample size. No interaction was observed between NLR and eGFR with p for interaction 0.94 and 0.41 for all-cause and CVD mortality.

### Sensitivity analyses

The sensitivity analyses, which excluded patients who died within 2 years (Supplementary Table S1) and those with an eGFR of less than 15 mL/min/1.73 m^2^ at baseline examination (Supplementary Table S3), produced consistent results for both all-cause and CVD mortality. To determine whether the prognostic signal is driven predominantly by neutrophilia, lymphopenia, or both, we present the independent associations of absolute neutrophil and lymphocyte counts with mortality outcomes in Supplementary Table S3.

### ROC analysis of the predictive value of the NLR

**FIGURE 6:**
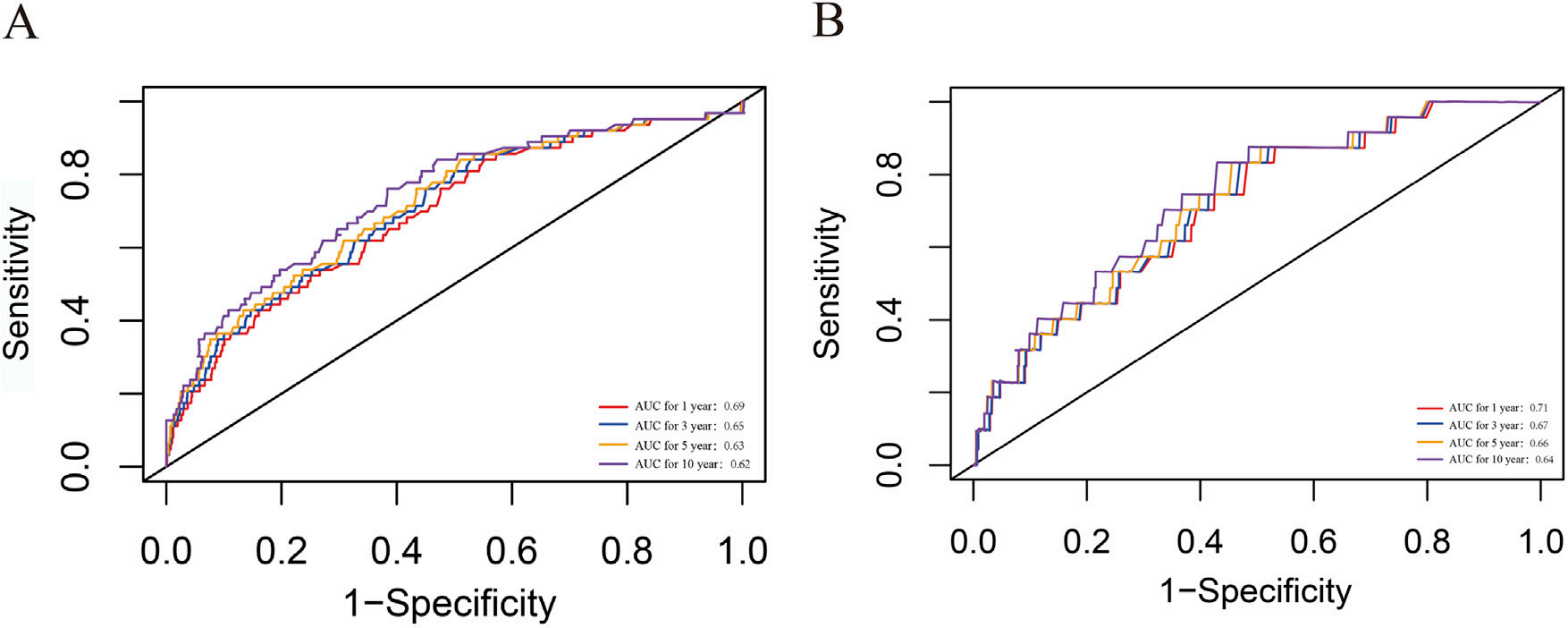
ROC curves of NLR in predicting 1-, 3-, 5-, and 10-year all-cause and CVD mortality. **(A)** all-cause mortality; **(B)** CVD mortality. CVD, cardiovascular disease; CKD, chronic kidney disease; NLR, Neutrophil-to-lymphocyte ratio; ROC, receiver operating characteristic curve.

The time-dependent ROC curve revealed that the areas under the curve for 1-, 3-, 5-, and 10-year survival rates were 0.69, 0.65, 0.63, and 0.62 for all-cause mortality, and as 0.71, 0.67, 0.66, and 0.64 for CVD mortality, respectively (Figure 6). Moreover, as detailed in Supplementary Table S4, integrating NLR alongside age and gender significantly strengthens its predictive capability. These findings suggest that the NLR possesses significant predictive power for both all-cause and CVD mortality in the short and long term and should be integrated into routine risk assessment for chronic kidney disease patients.

## Discussion

The study from the Global Burden of Disease project indicate that CKD is now a primary contributor to global mortality rate (Lousa et al., 2023), largely due to the ineffectiveness and/or untimeliness of traditional parameters in accurately reflecting the severity and prognosis of CKD. Given the NLR severing as a novel prognosis biomarker, the present study leveraged data from the NHANES cohort, spanning from 2009 to 2018, to elucidate the relationship between NLR and the risk of all-cause and CVD mortality in patients with CKD.

The mechanisms driving increased mortality in CKD are multifaceted and involve intricate pathophysiological alterations. A pivotal role in this process is played by heightened inflammation and immune dysfunction (Souza et al., 2015). Extensive research has shown that patients with CKD exhibit elevated levels of inflammatory mediators, such as high-sensitivity C-reactive protein, interleukin IL-6, and tumor necrosis factor-α (Kim et al., 2023; Zhang et al., 2017; Gao et al., 2023). These mediators stimulate the activation of mesangial, endothelial, and fibroblast cells, triggering the production of excessive extracellular matrix (Yoshitomi et al., 2019). This, in turn, leads to glomerular hypertension, tubulointerstitial fibrosis, and renal scarring. Furthermore, inflammation is crucial in the initiation and progression of atherosclerosis, a significant contributor to cardiovascular mortality, by facilitating leukocyte adhesion and infiltration into the vascular endothelium (Mahalle et al., 2013). Inflammation may promote the formation of atherosclerotic plaques by activating the immune system and recruiting inflammatory cells to the vessel wall (Cobeta et al., 2022). Moreover, it can enhance platelet activation and thrombus formation, leading to myocardial infarction or stroke (Adili et al., 2018). Furthermore, inflammatory cytokines may induce endothelial dysfunction and abnormal vascular tone (Kerget et al., 2019), thereby increasing the risk of hypertension and other cardiovascular outcomes. Consequently, investigating novel inflammatory markers for CKD patients could provide valuable insights into evaluating their mortality risk.

NLR, a readily accessible biomarker, has been previously established as an indicator of systemic inflammation. Previous studies have demonstrated a strong association between NLR and inflammatory markers such as TNF-α, CRP, and albumin, suggesting that high NLR reflects chronic inflammatory conditions in CKD patients (Malhotra et al., 2015; Turkmen et al., 2012). Moreover, recent advances reveal that peripheral lymphocytes exert protective effects and enhance cellular functions (Nishijima et al., 2015). In contrast, neutrophils contribute to pathogenesis by elevating MMP-9 levels, amplifying systemic inflammation, and generating free oxygen radicals—processes implicated in renal dysfunction and poorer clinical outcomes (Yamamoto et al., 2012). More and more studies have begun to explore the potential of NLR in evaluating the outcome status of CKD patients. The research conducted by Xin An (An et al., 2012) underscores the significance of NLR as a robust predictor of both overall and cardiovascular mortality among peritoneal dialysis patients. Similarly, Yalcin Solak (Solak et al., 2013) highlights the independent association between NLR and endothelial dysfunction, further emphasizing its potential to predict composite cardiovascular outcomes, even when accounting for traditional risk factors, in patients with moderate to severe CKD. Tomoko Abe (Abe et al., 2015) reinforces this notion by demonstrating that an elevated NLR is not only linked to a heightened risk of cardiovascular disease events but also serves as a more potent prognostic indicator for future events compared to other factors. These findings collectively emphasize the importance of NLR as a valuable biomarker in assessing and predicting cardiovascular risk in CKD patients. However, most of the previous studies only focused on end-stage or dialysis patients or with relatively small sample size. We performed an inaugural large-scale, nationally representative study to examine the correlation between NLR and the prognosis of CKD patients based on NHANES debase. Our findings indicate that a higher NLR is associated with an elevated risk of all-cause and CVD mortality in patients with CKD, even after accounting for potential confounding factors and excluding those with ESRD.

Our analyses further revealed a non-linear relationship between NLR and the HRs, with the overall risks of all-cause and CVD mortality escalating as NLR levels rise. Our study suggests that a specific range of NLR level should be determined to manage the mortality risk for CKD patients. Furthermore, taking into account the variation in NLR levels across different populations, we conducted RCS models separately for males and females to elucidate the dose-response relationship with mortality outcomes. Our results revealed that men are more sensitive than women and will be challenged with higher mortality risk as the NLR value increases, indicating different NLR control goals for men and women.

Additionally, it is crucial to acknowledge that NLR levels can be influenced by a multitude of factors, including gender, age, ethnicity, and BMI. To gain a deeper understanding of how NLR impacts the prognosis of CKD patients across diverse populations, we conducted a comprehensive subgroup analysis. The results of these analyses consistently pointed towards a relationship between NLR and CKD prognosis, though statistical significance was not achieved in all subgroups. The interaction analysis uncovered a significant interaction between BMI and NLR in relation to CVD mortality, suggesting that the impact of NLR on CKD prognosis is modulated by BMI. Considering that the various stages of renal function significantly influence the prognosis of CKD, we pay more attention to the relationship between eGFR and NLR. No significant interaction was detected between the NLR and eGFR for all-cause mortality and CVD mortality, suggesting that variations in renal function do not affect the relationship between NLR and the prognosis of patients with CKD. NLR significantly increases the death risk for G3a and G3b, with heightened risk in stage G3b, while the result of G4 and G5 phases are not statistically significant due to the small sample size with only 175 and 99 participants. Larger prospective studies are warranted to validate these findings for G4/G5 populations. Our findings imply that NLR management should be more nuanced and tailored to individual CKD patients, taking into account their diverse populations and characteristics, particularly across different BMI and eGFR stages.

To robust our conclusion, we further performed a sensitive analysis by excluding patients who died within 2 years and the end stage CKD patients. The result is almost consistent with the whole population indicating that NLR can be a generalized risk factor for CKD prognosis. Furthermore, the NLR demonstrated robust performance in predicting survival outcomes when assessed using time-dependent ROC curves. Specifically, it exhibited notable accuracy in forecasting 1-year all-cause mortality (AUC 0.69) and CVD mortality (AUC 0.71) in all participants. Our findings indicate that, in the management and monitoring of CKD patients, it is imperative to consider the NLR level alongside routine kidney function indicators such as eGFR, creatinine, and urinary protein. It is advisable to combine NLR with other biomarkers in clinical practice to enhance predictive accuracy.

Our findings not only reinforce the established correlation between elevated NLR and increased risk of CVD and all-cause mortality but also demonstrate its relevance across diverse subgroups of CKD patients, highlighting the potential for personalized risk assessment and management strategies. The application NLR as a prognostic tool in CKD patients also opens up possibilities for targeted therapeutic interventions. If NLR serves as an indicator of the systemic inflammatory status of CKD patients, then modulating this ratio might prove beneficial. For instance, strategies to reduce neutrophil activation or increase lymphocyte count could be explored. While this approach is largely speculative at this point, our findings provide a foundation for such explorations in future research.

While this study offers valuable insights into the potential association between NLR levels and CKD, it is important to acknowledge several key limitations that must be considered when interpreting the results. Firstly, as an observational study, our findings cannot conclusively establish causality between NLR levels and CKD, despite the large sample size and extended follow-up period. Secondly, the reliance on a single measurement of NLR levels within the NHANES dataset represents a significant limitation. This single snapshot in time may not accurately reflect an individual’s long-term NLR profile, which could be influenced by a variety of non-renal conditions such as acute infection, malignancy, systemic inflammation, and corticosteroid use, inevitably introducing potential bias. To mitigate this issue, future studies might consider incorporating multiple measurements of NLR levels over time and exclude potential confounders to better capture fluctuations and assess their impact on CKD outcomes. Nonetheless, single measurements of NLR levels have been reported in many large-scale cohort studies (Lan et al., 2023; Dong et al., 2023). Thirdly, the NHANES data lacks differentiation among the various underlying causes of CKD. Inflammation profiles differ substantially between conditions like diabetic nephropathy, hypertensive damage, and glomerulonephritis. Diabetic Nephropathy shows constant low-grade inflammation from metabolic dysfunction (Howlader et al., 2021), Glomerulonephritis exhibits episodic systemic spikes during autoimmune flares, and Hypertensive damage causes contained intrarenal oxidative stress. This heterogeneity in CKD etiology can significantly impact disease progression, treatment response, and ultimately alter how NLR predicts outcome. Besides, NHANES lacks longitudinal follow-up measurements to distinguish chronic kidney disease from transient or acute kidney injury. NLR-based survival predictions require careful interpretation and require future studies with longitudinal data to validate our observations. Despite these limitations, the complex multilevel sampling method employed by the NHANES provides a unique opportunity to generate findings that are likely more representative of the general population with CKD.

## Conclusion

Our study reveals compelling evidence of a robust association between an elevated NLR and an increased risk of CVD and all-cause mortality among patients with CKD. This finding underscores the potential of NLR as a valuable prognostic indicator for CKD patients, offering clinicians a simple yet powerful tool to assess disease severity and predict adverse outcomes.

## Data Availability

The original contributions presented in the study are included in the article/Supplementary Material, further inquiries can be directed to the corresponding authors.

## Abbreviations

CVD, cardiovascular disease; CKD, chronic kidney disease, NHANES, National Health and Nutritional Examination Survey; RCS, restricted cubic spline; HR, hazard ratio; CI, confidence intervals; eGFR, estimated glomerular filtration rate; ESRD, end-stages renal disease; NDI, National Death Index; CKD-EPI, Chronic Kidney Disease Epidemiology Collaboration; BMI, Body Mass Index; NLR, neutrophil-to-lymphocyte ratio; ROC, Receiver Operating Characteristic.
