# Supplementary material for: The role of neutrophil-to-lymphocyte ratio in the prognosis of chronic kidney disease: insights from the NHANES cohort study
Source: Front Syst Biol. 2025 Oct 27;5:1656683. doi: 10.3389/fsysb.2025.1656683 (PMC12597963; doi:10.3389/fsysb.2025.1656683)
Supplement: Supplementary file 1 [file Table1.docx]

**Supplementary Table 1. Multivariable-adjusted analyses for the associations of NLR with all-cause and CVD mortality among CKD from NHANES 2009-2018 after excluding the patients died within two years**

|  |  | **HR (95% CI)** | | | |
| --- | --- | --- | --- | --- | --- |
|  | **No. of Events** | **Model 1** | **Model 2** | **Model 3** | **Model 4** |
| **All-cause mortality** |  |  |  |  |  |
| NLR (continuous) | 536 | 1.10 (1.04, 1.16) | 1.53 (1.23, 1.89) | 1.08(1.02, 1.13) | 1.07(1.07, 1.08) |
| NLR (categorical) |  |  |  |  |  |
| NLR_1 | 210 | 1.0(reference) | 1.0(reference) | 1.0(reference) | 1.0(reference) |
| NLR_2 | 326 | 1.80(1.46, 2.24) | 1.53 (1.23, 1.89) | 1.50(1.21,1.85) | 1.51(1.47,1.54) |
| **CVD mortality** |  |  |  |  |  |
| NLR (continuous) | 202 | 1.11 (1.05, 1.18) | 1.97 (1.1.37, 2.83) | 1.09 (1.03, 1.16) | 1.08(1.08, 1.09) |
| NLR (categorical) |  |  |  |  |  |
| NLR_1 | 70 | 1.0(reference) | 1.0(reference) | 1.0(reference) | 1.0(reference) |
| NLR_2 | 132 | 2.38(1.68,3.39) | 1.96(1.37,2.83) | 1.95(1.34,2.83) | 1.76(1.69,1.82) |

Values are n or weighted HR (95% CI). Model 1 is unadjusted; Model 2 is adjusted for: Age, Sex and Race; Model 3 is adjusted for: model 2 + Alcohol intake, Smoking status, BMI, Ratio of family income to poverty, and Education level; Model 4 is adjusted for: model 3 plus Diabetes, Hypertension, Dyslipidemia, and eGFR; CVD, cardiovascular disease; CKD, chronic kidney disease, NHANES, National Health and Nutritional Examination Survey; NLR, Neutrophil-to-lymphocyte ratio.
