# Supplementary material for: The role of neutrophil-to-lymphocyte ratio in the prognosis of chronic kidney disease: insights from the NHANES cohort study
Source: Front Syst Biol. 2025 Oct 27;5:1656683. doi: 10.3389/fsysb.2025.1656683 (PMC12597963; doi:10.3389/fsysb.2025.1656683)
Supplement: Supplementary file 2 [file Table2.docx]

**Supplementary Table 2. Multivariable-adjusted analyses for the associations of NLR with all-cause and CVD mortality among CKD from NHANES 2009-2018 after excluding the patients with eGFR<15 mL/min/1.73 m^2^**

|  |  | **HR (95% CI)** | | | |
| --- | --- | --- | --- | --- | --- |
|  | **No. of Events** | **Model 1** | **Model 2** | **Model 3** | **Model 4** |
| **All-cause mortality** |  |  |  |  |  |
| NLR (continuous) | 717 | 1.18 (1.14, 1.22) | 1.17 (1.12, 1.21) | 1.15(1.11, 1.20) | 1.12(1.07, 1.17) |
| NLR (categorical) |  |  |  |  |  |
| NLR_1 | 273 | 1.0(reference) | 1.0(reference) | 1.0(reference) | 1.0(reference) |
| NLR_2 | 444 | 1.87(1.55, 2.26) | 1.76 (1.46, 2.13) | 1.64(1.36,1.98) | 1.54(1.28,1.86) |
| **CVD mortality** |  |  |  |  |  |
| NLR (continuous) | 262 | 1.19 (1.14, 1.26) | 1.18 (1.12, 1.25) | 1.17 (1.10, 1.24) | 1.14(1.07, 1.22) |
| NLR (categorical) |  |  |  |  |  |
| NLR_1 | 85 | 1.0(reference) | 1.0(reference) | 1.0(reference) | 1.0(reference) |
| NLR_2 | 177 | 2.58(1.88,3.52) | 2.41(1.76,3.30) | 2.28(1.65,3.14) | 2.15(1.56,2.96) |

Values are n or weighted HR (95% CI). Model 1 is unadjusted; Model 2 is adjusted for: Age, Sex and Race; Model 3 is adjusted for: model 2 + Alcohol intake, Smoking status, BMI, Ratio of family income to poverty, and Education level; Model 4 is adjusted for: model 3 plus Diabetes, Hypertension, Dyslipidemia, and eGFR; CVD, cardiovascular disease; CKD, chronic kidney disease, NHANES, National Health and Nutritional Examination Survey; NLR, Neutrophil-to-lymphocyte ratio.
