# Supplementary material for: The role of neutrophil-to-lymphocyte ratio in the prognosis of chronic kidney disease: insights from the NHANES cohort study
Source: Front Syst Biol. 2025 Oct 27;5:1656683. doi: 10.3389/fsysb.2025.1656683 (PMC12597963; doi:10.3389/fsysb.2025.1656683)
Supplement: Supplementary file 3 [file Table3.docx]

**Supplementary Table 3. Association of Neutrophils, Lymphocytes,**

**and NLR with Prognosis in CKD Patients.**

|  | **HR (95% CI)** | **P-value** | **AUC for 10 years survival** |
| --- | --- | --- | --- |
| **All-cause mortality** |  |  |  |
| Neutrophils number | 1.07(1.05, 1.09) | <0.001 | 0.55 |
| Lymphocyte number | 1.00(0.99, 1.02) | 0.6 | 0.42 |
| NLR (continuous) | 1.04(1.02, 1.14) | <0.001 | 0.62 |
| **CVD mortality** |  |  |  |
| Neutrophils number | 1.06 (1.03, 1.09) | <0.001 | 0.56 |
| Lymphocyte number | 0.82(0.59, 1.15) | 0.3 | 0.39 |
| NLR (continuous) | 1.10(1.04, 1.16) | <0.001 | 0.64 |

Model is adjusted for Age, Sex , Race, Alcohol intake, Smoking status, BMI, Ratio of family

income to poverty, Education level, Diabetes, Hypertension, Dyslipidemia, and eGFR.

CVD, cardiovascular disease; CKD, chronic kidney disease; NLR, Neutrophil-to-lymphocyte ratio.
