# Supplementary material for: The role of neutrophil-to-lymphocyte ratio in the prognosis of chronic kidney disease: insights from the NHANES cohort study
Source: Front Syst Biol. 2025 Oct 27;5:1656683. doi: 10.3389/fsysb.2025.1656683 (PMC12597963; doi:10.3389/fsysb.2025.1656683)
Supplement: Supplementary file 4 [file Table4.docx]

**Supplementary Table 4. Improvement in AUC When Adding NLR**

**to Age and Gender in predicting 1-,3-, 5-, and 10-year all-cause**

**and CVD mortality .**

|  | **AUC for survival** | | | |
| --- | --- | --- | --- | --- |
|  | **1 years** | **3 years** | **5 years** | **10 years** |
| **All-cause mortality** |  |  |  |  |
| age+gender | 0.44 | 0.51 | 0.54 | 0.57 |
| NLR | 0.69 | 0,65 | 0.63 | 0.62 |
| NLR+age+gender | 0.68 | 0.71 | 073 | 0.72 |
| **CVD mortality** |  |  |  |  |
| age+gender | 0.60 | 0.66 | 0.71 | 0.71 |
| NLR | 0.71 | 0.67 | 0.66 | 0.64 |
| NLR+age+gende | 0.64 | 0.69 | 0.75 | 0.73 |

CVD, cardiovascular disease; NLR, Neutrophil-to-lymphocyte ratio; Area Under the

ROC Curve; ROC, receiver operating characteristic curve.
